# FEM-Based Conductive Heat Transfer Analytical Description of Solidification Rate and Temperature Gradient during Lateral Laser Beam Oscillation Welding of Aluminum Alloy

**DOI:** 10.3390/ma17133248

**Published:** 2024-07-02

**Authors:** Jason Cheon, Cheolhee Kim, Sanghoon Kang, Minjung Kang

**Affiliations:** 1 Flexible Manufacturing R&D Department, Korea Institute of Industrial Technology, Incheon 21999, Republic of Korea; jasonhp@kitech.re.kr (J.C.); y2khyh3043@kitech.re.kr (S.K.); 2 Department of Mechanical and Materials Engineering, Portland State University, Portland, OR 97201, USA; cheol@pdx.edu; 3 School of Mechanical Engineering, Yonsei University, Seoul 03722, Republic of Korea

**Keywords:** laser beam oscillation welding, aluminum, conductive heat transfer analysis, solidification rate, temperature gradient, microstructure formation

## Abstract

This study investigates the feasibility of utilizing the finite element method (FEM)-based conductive heat transfer (CHT) analysis simulation to determine temperature gradients and solidification rates at the solid–liquid interface during laser beam oscillation welding. By comparing experimental observations with FEM-based CHT analysis, the underlying microstructural evolution and grain formation during welding were examined. FEM-based CHT enables the calculation of temperature gradients (*G*) and solidification rates (*R*), offering insights into the formation of equiaxed structures, which are crucial for suppressing hot cracking. Columnar-to-equiaxed structure transition thresholds, such as *G/R* and *G*^3^/*R*, accurately predict the emergence of fully equiaxed grain structures, validated by electron backscatter diffraction. This research provides valuable insights into temperature gradients and solidification rates in oscillation welding, guiding process design for achieving refined equiaxed structures and minimizing hot cracks.

## 1. Introduction

The electric vehicle industry prioritizes developing lightweight body-in-white and battery components using aluminum alloys, particularly the 6xxx series alloy, due to their excellent strength-to-weight ratio and corrosion resistance [1,2,3]. However, suppressing solidification cracking in aluminum welds is challenging. The low ductility of the semi-solid in the mushy zone, coupled with the high solidification shrinkage of aluminum alloys, considerably increases susceptibility to hot cracking [4,5]. Solidification cracking is initiated by complex interactions between metallurgical and mechanical factors [6]. During laser welding, these issues can be mitigated by improving the chemical composition, refining the solidification structure, optimizing laser pulsing parameters, and/or reducing thermal strains [7].

The laser beam oscillating welding (LBOW) technique has been widely adopted for its outstanding abilities in gap bridging, porosity, and crack suppression [8,9,10,11,12]. In addition, LBOW promotes fine equiaxed dendritic grain structure, offering superior resistance to hot cracking and improved mechanical properties compared to elongated columnar dendritic grains [13]. This is because the oscillation of the heat source influences heat flow and microstructure formation [14]. Laser beam oscillation regulates factors impacting microstructural evolution and thermal strain, such as the behavior of the molten pool, temperature gradient, and solidification rate [14]. Komela et al. [15] analyzed laser weld beads and solidified morphology, observing equiaxed or columnar dendrites based on oscillation parameters. Wang et al. [16] obtained a fine equiaxed microstructure at the center of the fusion zone, aided by the abundant nuclei supplied by the oscillated molten pool. Hagenlocher et al. [17] explored beam oscillation effects on local solidification rate and temperature gradient using a pyrometer and high-speed camera. They reported that a high volume of equiaxed structure can be obtained by inducing oscillation welding. Therefore, it is recognized that heat transfer and molten pool flow are crucial for welding quality and performance [18]. However, accurately evaluating solid–liquid interface temperature via experimental methods (such as pyrometers and thermal cameras) remains challenging, as the measurement spot size is larger compared to the object.

In laser welding, the high density of the focused laser beam is irradiated onto the substrate, where complicated phenomena such as the time-temperature dependency phase transition (i.e., melting and evaporation) occur in a short time. Numerical simulation is a convenient and efficient method to reveal the underlying physics during the welding process. However, previous research on simulation for LBOW has focused on insights into heat transfer, fluid flow, and stress distribution surrounding the welds. Chukkan et al. [19] carried out a thermo-elasto-plastic analysis during laser beam welding using three different heat sources. They simulated thermal cycles, residual stresses, and distortion and compared them with experimental results. Bu et al. [20] simulated the laser wobbling joining of thermoplastic and Ti-6Al-4V alloy using a thermo-mechanically coupled model and discussed the thermal distribution and residual stress characteristics.

Ai et al. [21,22] developed a computational fluid dynamics (CFD) model using the finite volume method for finite-shaped oscillating laser welds to discuss the characteristics of bead shape, temperature field, and fluid flow field in the molten pool. They found that the temperature gradient and velocity of the molten pool decrease as the oscillation amplitude or frequency increases. Ai et al. [23] developed a microscale phase field model to predict the microstructure of welds formed under circular-shaped oscillating laser beams and compared the primary dendrite arm spacing at different laser powers, speeds, oscillation amplitudes, and frequencies. They observed that the temperature gradient decreases rapidly, and the melt flow rate increases significantly with an oscillating laser. Wu et al. [24] observed the molten pool dynamics during laser additive manufacturing using an in-situ high-speed synchrotron X-ray radiography and using the numerical simulation to analyze the melt and gas flows. Pamarthi et al. [25] employed a monitoring methodology to examine the solidification cracking in partially penetrated welds using quartz glass. Lu et al. [18] numerically analyzed the heat transfer and melt flow in the circular-shaped oscillation welding process. The simulation of the laser welding process enables the estimation of temperature gradient and solidification rate, which determine the microstructural formation. Additionally, Zhang et al. [26] performed a CFD simulation and revealed that the temperature gradient was reduced from 279 to 38.3 K/mm, and the molten pool flow rate increased from 0.515 to 0.998 m/s as the oscillation frequency changed from 30 to 90 Hz. These results showed that bead shape, temperature field, molten pool flow rate, and microstructure formation were significantly affected by the oscillation condition. The information from the temperature field is abundant in calculating crack sensitivity-related parameters.

Finite element method (FEM)-based conductive heat transfer (CHT) analysis can quickly and simply derive the temperature field and is considered easier than CFD-based calculations that simultaneously solve the temperature, velocity, and pressure fields. In applications requiring multiple physical values, such as temperature, pressure, velocity, and the shape and size of the free surface, CFD simulation is an appropriate approach. However, it requires coupled calculations among the continuity, momentum, and energy equations, as well as at least one governing equation for free surface tracking. Additionally, each equation requires physical or mathematical assumptions and related material properties. This complexity can sometimes lead to uncertainty in the solution and a significant gap between calculation results and real physical behaviors. An approach with FEM-based CHT analysis also has uncertainty problems arising from physical assumptions and related properties. However, since it was proposed by Goldak in 1984 [27], this method has been widely used and improved to calculate the thermal field only. Its application for welding process reproduction rather than prediction has been verified through many existing studies, confirming its reliability.

This study aims to demonstrate the potential of employing FEM-based CHT simulation for an analytical description of temperature gradients and solidification rates at the solid–liquid interface, particularly focusing on the molten pool boundary. Through a combination of experimental observations and FEM-based CHT analysis, the underlying mechanisms governing microstructural evolution and grain formation on the molten pool boundary are discussed. The simulation model facilitates the determination of temperature gradients (*G*) and solidification rates (*R*) at the fusion boundary, providing crucial insights into the formation of equiaxed structures. Through this analysis, we aim to investigate the impact of beam oscillation on molten pool dynamics and predict the localized formation of equiaxed microstructure throughout an oscillation cycle.

## 2. Experimental Procedure

### 2.1. Laser Beam Oscillation Welding

For the LBOW, a continuous-mode fiber laser, YLS-6000 (IPG Photonics, Oxford, MA, USA), was used with a two-axis scanner, D30 (IPG Photonics, Oxford, MA, USA). The scanner had a focal length of 200 mm and delivered the laser beam perpendicularly to the specimen. The measured diameter of the focused beam was 0.27 mm on the surface of the workpiece. Laser welding was performed on a bead-on-plate without shielding gas. The base material was a 1 mm-thick Al 6014-T4 alloy sheet.

**Figure 1:**
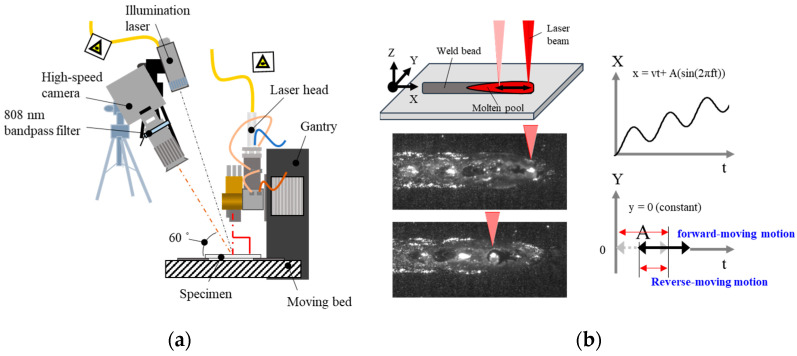
(**a**) Schematic of the experimental setup and (**b**) baser beam movement within a cycle.

During welding, a high-speed camera (Mini UX30, PHOTON, Tokyo, Japan) recorded the molten pool surface with a resolution of 896 × 488 pixels, a sampling rate of 5000 fps, and a 1/fps shutter speed. The high-speed camera was tilted at an angle of 60° relative to the specimen. Additionally, an illumination laser (ILS LIMO120-F400, LIMO, Dortmund, Germany) was installed to obtain clear images. The wavelength and power of the illumination laser were 808 nm and 30 W, respectively. A bandpass filter with an 808 ± 5 nm band was attached in front of the high-speed camera, as depicted in Figure 1.

**Figure 2:**
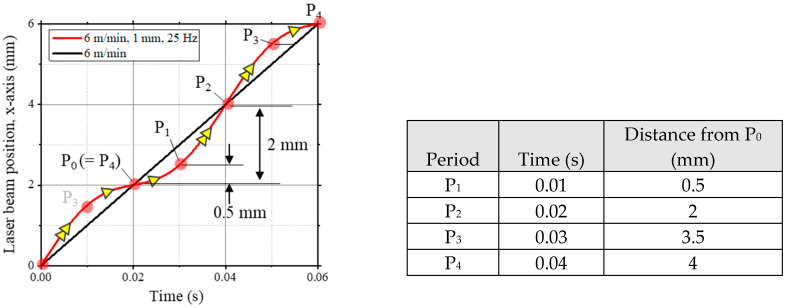
X-coordinate changes of oscillated laser beam and typical linear laser beam pattern.

The laser beam migrated according to a lateral moving pattern, with the oscillation width and frequency fixed at 1 mm and 25 Hz, respectively. Laser weld parameters are summarized in Table 1. The laser beam laterally transversed forward and backward into the molten pool according to the oscillation frequency and width (Figure 1b). To explain the sequential behavior of the laser source, the periodic movement of the laser beam was described as four periods with 0.001 s of time intervals, as P_1_ to P_4_. By combining travel speed and beam path movement, the velocity of the laser beam from P_1_ to P_3_ was accelerated, while from P_3_ to P_1_, the laser beam had a lower speed. During P_1_ to P_3_, the total moving distance was 3 mm, which is three times higher than that of the P_3_ to P_1_ period. The relative laser beam position compared to typical linear welding is presented in Figure 2.

After welding, the transversely and horizontally sectioned specimens were polished and examined for microstructure observation and macroscopic measurement of the fusion zone shape. The structure of the welds was analyzed via optical microscopy and field-emission scanning electron microscopy (Inspect F50, FEI Company, Hillsboro, OR, USA) equipped with electron backscatter diffraction (EBSD) (Hikari EBSD, AMETEK, Berwyn, PA, USA). The EBSD specimens were mechanically polished and then electro-polished at room temperature in a solution of 10% perchloric acid and ethanol. The high-angle grain boundary was set at 15° for grain identification.

### 2.2. Computation Model

**Figure 3:**
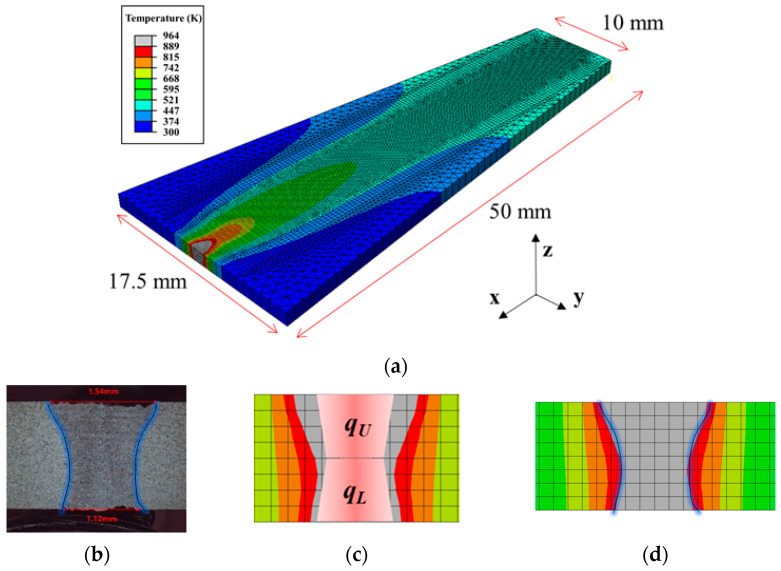
(**a**) Model geometry used in the simulation, (**b**) macro-section of the welded sample, (**c**) schematic of dual conical heat source, and (**d**) sectional image of simulation results. The blue lines of (**b**,**d**) were extracted from the fusion zone shape on macro-section and the gradated red trapezoids of (**c**) refer volume of dual conical heat source.

The finite element model was developed using the commercial software Abaqus/Standard (Ver. 2022) with a thermal analysis type. The model geometry mimicked the self-restrained test coupon used in a previous study by the author [28] for a hot cracking test (Figure 3a).

To emulate LBOW, vertically arranged and simultaneously moving dual conical volumetric heat sources were utilized (Figure 3c). A conical volumetric heat source formula [29,30] was applied as follows:

(1) $${\dot{q}}_{i}^{\prime \prime \prime }=\frac{1}{2}\frac{\delta 9\eta Pexp(3)}{\pi (\exp\left(3\right)-1)}\cdot \frac{1}{C_{i}}exp(-3(\frac{x^{2}+y_{i}^{2}}{r_{i0}^{2}}))$$

(2) $$C_{i}=\left(z_{iT}+z_{iB}\right)\left(r_{iT}^{2}+r_{iB}^{2}+r_{iT}r_{iB}\right)$$

(3) $$r_{i0}=r_{iT}-\frac{\left(r_{iT}-r_{iB})(z_{iT}-z_{i}\right)}{z_{iT}-z_{iB}}$$

where *P* and *η* are the laser power and process efficiency, respectively, with *η* set to 0.24. The subscript *i* refers to the upper heat source, *q_U_*, and lower heat source, *q_L_*, respectively. The heat fraction of *q_U_* and *q_L_* is 1:1. *x* represents a moving coordinate combining welding speed and oscillation beam parameter. *r_ij_* and *z_ij_* are the radii and heights of the heat source, respectively. The subscript *j* represents the location of the heat source, with T and B indicating the top and bottom heat sources, respectively. The minimum mesh size was set to 0.2 mm × 0.2 mm × 1.875 mm to ensure convergence. *r_ij_* and *z_ij_* were tuned by trial and error to align the simulation with reality, as evidenced by the simulated fusion line, showing a close match with the experimental result (Figure 3b,d). The physical and thermo-mechanical properties used in the model are presented in Table 2.

The movement of the volumetric heat source with lateral motion in the *z* direction is described as follows:

(4) $$x=\left(x_{o}+Vt\right)+A\times sin\left(2\pi f\times t\right)$$

where *V* is speed, *t* is time, *A* is oscillation width, and *f* is frequency.

Solving the following conductive energy equation allows us to derive the temperature history and distribution:

(5) $$\rho C_{p}\frac{\partial T}{\partial t}=\frac{\partial }{\partial x}\left(k\frac{\partial T}{\partial x}\right)+\frac{\partial }{\partial y}\left(k\frac{\partial T}{\partial y}\right)+\frac{\partial }{\partial z}\left(k\frac{\partial T}{\partial z}\right)+{\dot{q}}^{\prime \prime \prime }$$

where *T*, *ρ*, *Cp*, and *k* are the temperature, density, specific heat, and CHT coefficient, respectively. To mimic the convective heat transfer effect, the *k* value for the liquid state was treated as three times the value of the ambient condition. Convective and radiative surface heat losses, based on Newton’s law of cooling, were factored in with a 15 W/m^2^K convective heat transfer coefficient and 0.2 surface emissivity.

## 3. Results

### 3.1. Molten Pool Behavior during Laser Welding and Microstructure Observation

**Figure 4:**
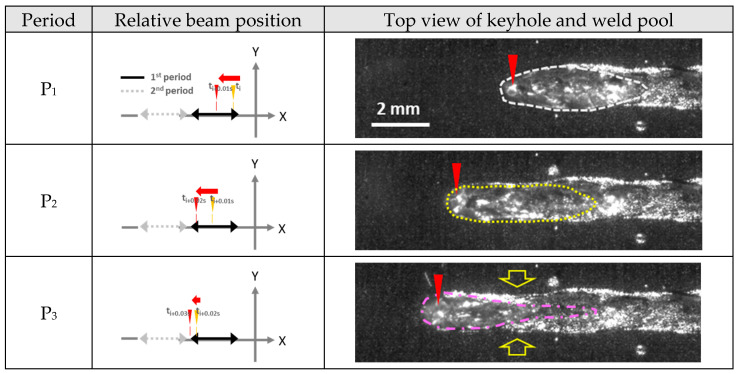

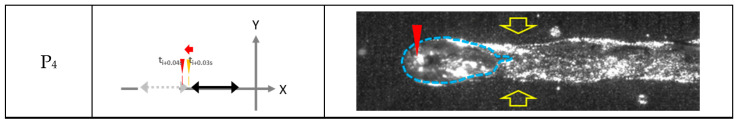
Molten pool development during lateral oscillation welding.

The shape of the molten pool changed periodically depending on the keyhole location, as shown in the high-speed camera images in Figure 4 (molten pool boundary indicated by colored dotted frame), with the location of the keyhole indicated by a red arrow. As the keyhole velocity accelerated (from P_1_ to P_3_), the length of the molten pool was elongated, and the shape of the molten pool tail became rounded. When the keyhole velocity was lowered (from P_3_ to P_1_), the molten area contracted sharply. The tail of the molten pool became sharper due to rapid solidification in the transverse direction. The necking of bead width was observed after P_3_ as a result of the solidification of the elongated molten pool (indicated by yellow arrows in Figure 4(P_3_,P_4_)). The length of the molten pool decreased sharply compared with the width of the molten pool.

**Figure 5:**
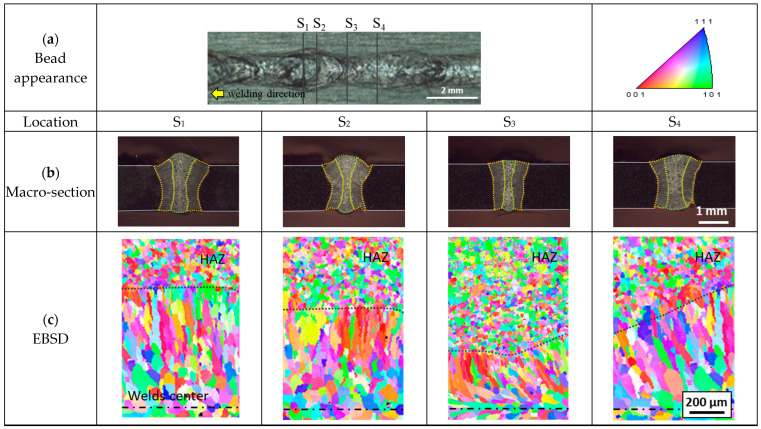
Observation of (**a**) bead appearance and microstructure, (**b**) optical microscope, and (**c**) EBSD.

The change in the shape of the tail indicated that the grain growth and the resulting structure could also be altered with the solid–liquid interface movement [13,33,34]. This is because the quantity of heat flow and its direction, as well as the grain at the solid–liquid interface, act as the substrate for nucleation in fusion welding. Bead appearance, macro-section, and EBSD images at different sectors are presented in Figure 5. An equiaxed structure was formed at the center of the fusion zone, whereas a columnar structure was generated along the boundary of the fusion line. The boundary between the heat affected zone and weld was marked using a a black dotted line. An inhomogeneous bead shape, molten area, and microstructural consistency were observed even within one cycle. In addition, the growth direction of the columnar grains also changed according to the period (Figure 5c). In S_1_ and S_2_, the growth direction of columnar grains was perpendicular to the welding direction, and in S_3_ and S_4_, the columnar grains developed in the opposite direction to the welding direction. The fraction of equiaxed structure was lowest at S_3_ and highest at S_1_ and S_4_.

### 3.2. Simulation of Laser Beam Oscillation Welding

**Figure 6:**
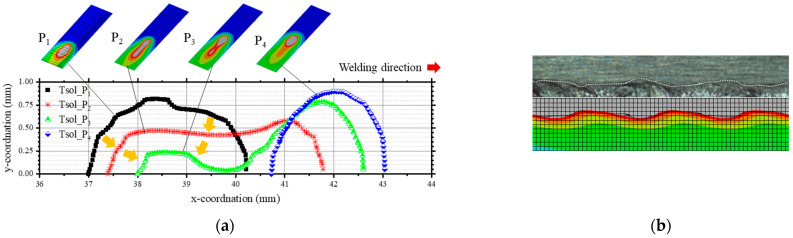
Temperature distributions: (**a**) on the surface of the workpiece in one cycle, and (**b**) predicted and actual fusion boundary comparison. The yellow arrow indicates the direction of the solid–liquid interface movement.

Calculated isothermal solidus lines, as depicted in Figure 6a, matched well with the molten pool geometry obtained using the high-speed camera from Figure 4. The part of the mesh that heated to above the melting temperature was reconstructed and compared with the actual bead appearance (Figure 6b). The calculated bead exhibited a similar fluctuating bead shape to reality.

**Figure 7:**
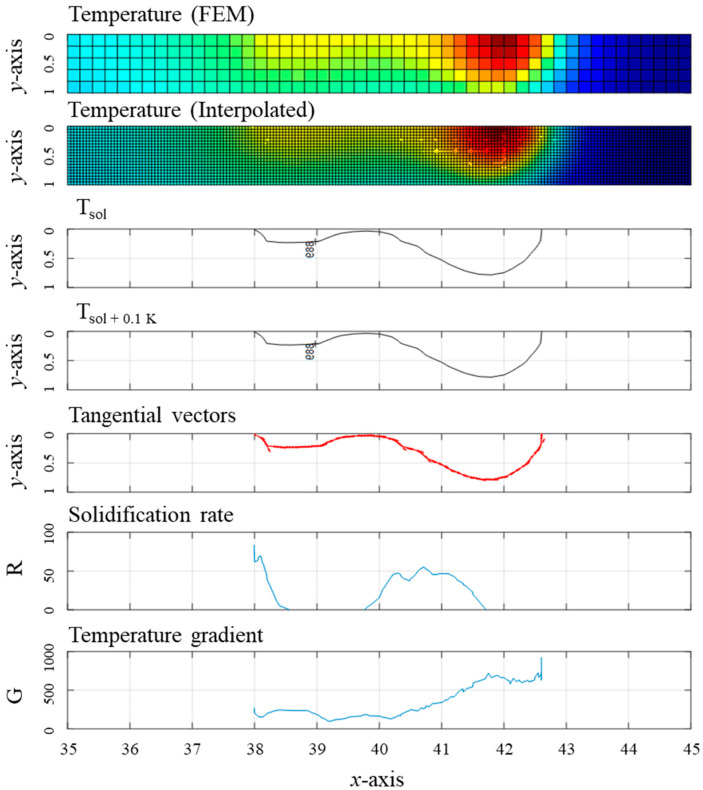
Example of the *G*- and *R*-value calculation (at P_3_).

As shown in Figure 7, the isothermal temperature lines, tangential vector line, and *G*- and *R*-values were extracted according to the oscillation period using the script developed by the author with Matlab^TM^. *G* was obtained as the multiplication of the temperature difference and the distance in the normal vector as follows:

(6) $$G=dT/ds=\left(T_{liq}-T_{sol}\right)/(|s|)$$

where *T_liq_* and *T_sol_* indicate the isothermal liquidus and solidus temperatures, respectively.

**Figure 8:**
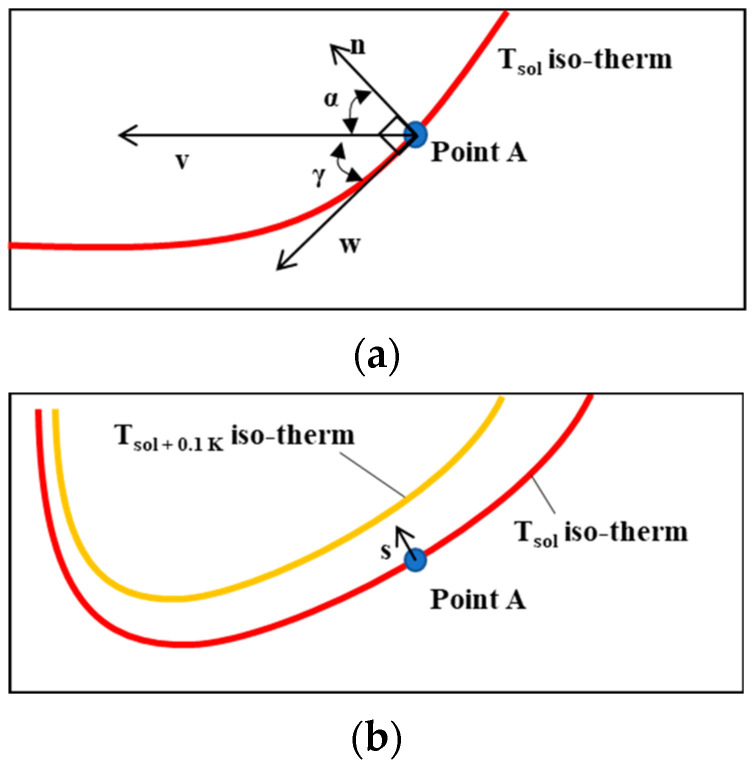
(**a**) Definition in temperature gradient *G* and (**b**) solidification rate *R* along the isothermal boundary.

Due to the complex geometry of the melting line, normal vector lines intersect severely when *T_liq_* and *T_sol_* are set as the target temperatures. Therefore, in this study, the temperature difference was calculated using a temperature close to the isothermal solidus temperature. Setting a target temperature with a small deviation assisted in extracting the precise shape of the isotherm temperature lines consistent with the molten pool, thereby preventing interference between each normal vector. In Figure 8b, the normal vector distance, |*s*|, was depicted as follows:

(7) $$|s|=\sqrt{\left[{\left(x_{sol}-x_{sol+0.1\;K}\right)}^{2}+{\left(y_{sol}-y_{sol+0.1\;K}\right)}^{2}\right]}$$

*R* is defined as *R* = *V* × cos (*α*) where *V* is the welding speed. To calculate *R*, the angle (γ) was extracted between the velocity vector (*v*) and the tangent vector (*w*) at a random node on the isothermal solidus line, as shown in Figure 8a. Angle *α* was then calculated from the equation *α* = π/2 − γ. *v* was set to aim at the maximum temperature point.

**Figure 9:**
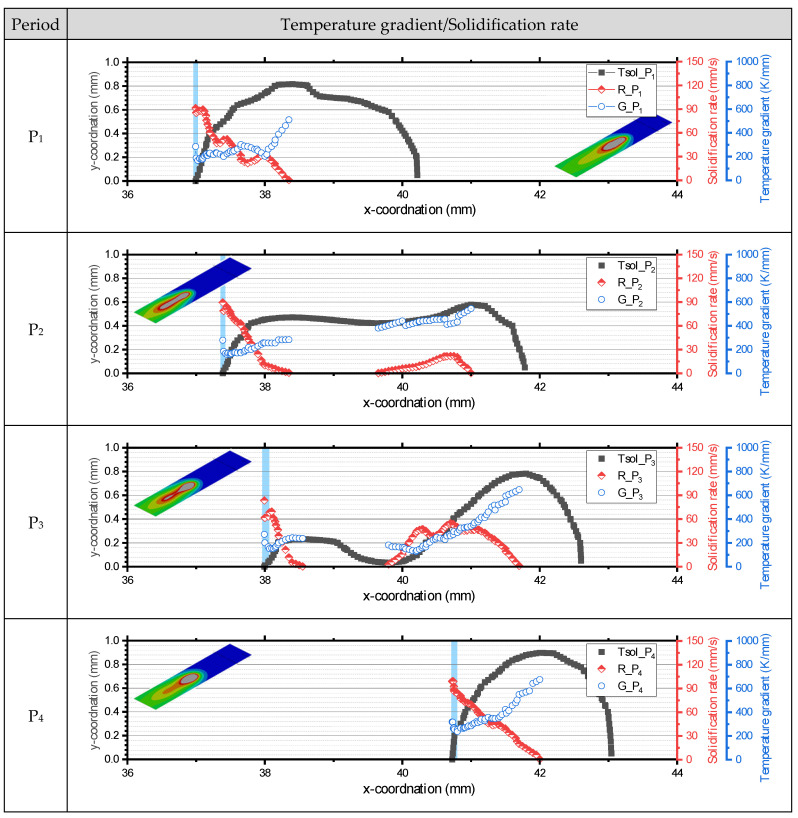
Local temperature gradient and solidification rate calculations.

The temperature gradient always has a positive value due to the nature of the equation, while the solidification rate can exhibit a negative value depending on the angle between the velocity and tangent vectors. Since a negative solidification rate implies re-melting, solidification rates in Figure 9 are expressed only as positive values.

The solidification rate and temperature gradient distribution changed depending on the oscillation period, as shown in Figure 9. At the weld centerline (y = 0, *α* = 0), the distance between the maximum pool temperature and the pool boundary is greater than at the fusion line (y = 0.8, *α* = 90) because the weld pool is elongated. As the *y*-axis approaches 0, *R* gradually increases, and *G* tends to decrease. At P_1_ and P_4_, *G* and *R* have continuous values; however, at P_2_ and P_3_, necking of the isothermal line separated the *G* and *R* curves.

The highest *R*-value was calculated at the tail of the molten pool, where the angle to the velocity vector was 0°. As the keyhole moved faster during P_1_ to P_3_, the calculated maximum *R*-value was approximately 90 mm/s, while the maximum *R*-value at P_4_ reached 100 mm/s. During P_1_ to P_3_, the calculated maximum *G*-value was less than 600 K/mm, while it surpassed 600 K/mm when the keyhole moved slower during P_3_ to P_1_. *G* sharply increased at the tail of the molten pool (vertical blue line in Figure 9), and the differences were greater from P_1_ to P_3_. Necking of the molten pool at P_2_ and P_3_ caused the breaking of the *R* and *G* curves. The rear of the molten pool has a relatively high *R*-value and low *G*-value compared to the front. The lowest *G*-value was calculated at P_3_, where the boundary followed the molten pool and the highest *R*-value was found at P_4_. It is inferred that there is a time delay between the keyhole movement and molten pool formation.

## 4. Discussion

In this study, LBOW with a 25 Hz oscillation frequency and 1 mm width was performed and emulated using FEM-based CHT analysis. The simulation model provided *G* and *R* at the fusion boundary, which allowed for the prediction of the formation of an equiaxed structure. In high-speed camera images (Figure 4), the molten pool stretched from P_1_ to P_3_ when the keyhole moved faster and then shrank. The FEM-based CHT analysis results show that the molten pool separated into the rear and front at P_3_ (Figure 6 and Figure 7), but the solid–liquid interface was not separated but narrowed in reality (Figure 4). However, the calculated bead appearance has a high consistency with the actual bead appearance.

The study demonstrates how the strong fluctuation of the molten pool influences the dynamics of microstructure distribution. In sections S_1_ and S_2_ in Figure 5, columnar grains are observed to grow perpendicular to the welding direction. Conversely, in sections S_3_ and S_4_, grains grow in the direction opposite to that of welding. This suggests that the elongation of the molten pool during faster keyhole movements creates a secondary local maximum temperature point distinct from the primary one at the laser focal point. Furthermore, the movement of the solidus boundary in the direction opposite to welding, especially noticeable behind the necking region (indicated by a yellow arrow in Figure 4 and Figure 6a), is observed. High-speed camera images corroborate this, showing the weld bead boundary solidifying while the center remains molten at P_3_.

**Figure 10:**
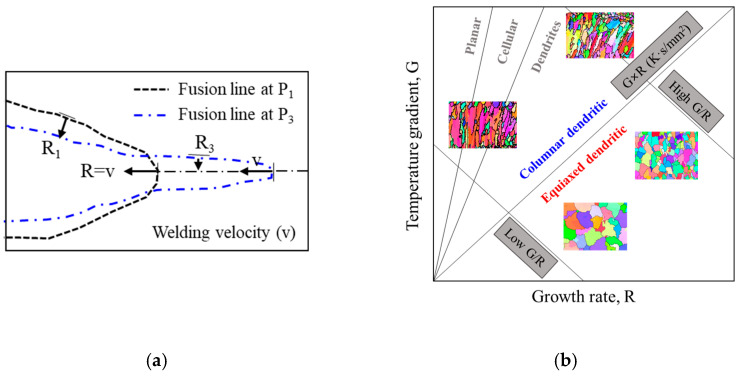
Schematic diagram of (**a**) solidification rate on the molten pool boundary and (**b**) solidification structure map.

The rapid changes in molten pool length caused by LBOW are expected to significantly affect *R*, as depicted in Figure 9. Additionally, *G* was influenced by laser beam oscillation, following the findings from previous studies by Ai et al. [21,22]. At isothermal steady-state conditions, *R* at the centerline of the weld corresponds to the welding velocity, while *R* at the boundary of the weld is relatively lower, as shown in Figure 10a. Compared to previous experimental studies, *G* and *R* calculated through the simulation were approximately twice as high. Hagenlocher et al. [17] conducted laser welding using different laser powers, patterns, and amplitudes at a welding speed of 4 m/min and measured *G* and *R* at the center under different welding oscillation conditions via a high-speed camera and two pyrometers. When the oscillation pattern changed from linear to sinusoidal at a welding speed of 4 m/min, the *G* at the centerline changed from 92 K/mm to 85–93 K/mm, and *R* was 66.67 mm/s, independent of the oscillation condition because the solid–liquid boundary is normal to the welding direction. The study by Schempp [35] exhibited a similar tendency. This discrepancy is attributed to the simulations not being conducted under the same welding parameters as the experimental results. In the experiments, the variation in the molten pool’s length was not severe, and the selected isotherm temperature had a wide divergence. Moreover, the targeting point where the pyrometer measured temperature was significantly larger than the nodes constituting the solid–liquid interface in the simulation.

Understanding the transition from columnar to equiaxed structure (CET) is crucial due to its impact on the size and distribution of equiaxed structures, influencing hot cracking behavior [36]. Figure 10b is a solidification structure map according to Kou [13], illustrating how *G* and *R* influence the resulting grain structure. The ratio *G/R* determines the mode of solidification, and *G×R* affects the size of the microstructure. Low *G* and high *R* lead to an equiaxed dendritic structure, while high *G* and low *R* result in oriented growth of columnar dendritic grains. Hagenlocher et al. [37] presented the CET threshold (*G/R*) as 3 Ks/mm^2^. Additionally, Geng et al. [38] introduced another CET criterion, *G*^3^/*R*, when it exceeded 1.66 × 10⁵ K^3^/mm⁴, indicating the occurrence of fully equiaxed grain formation.

**Figure 11:**
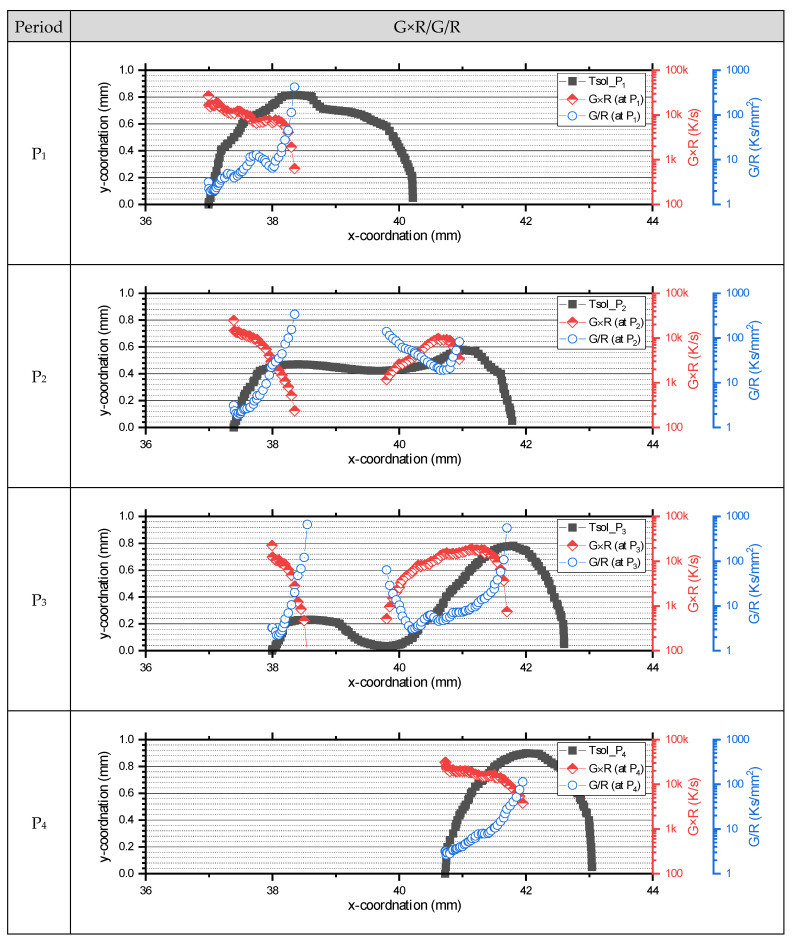
Calculated G×R and G/R throughout an oscillation cycle.

**Figure 12:**
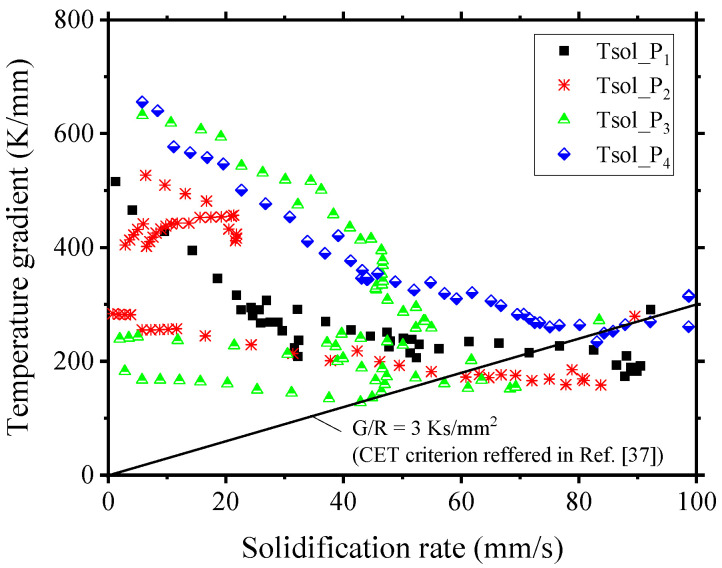
Re-constructed solidification structure map using simulation results [37].

The *G×R* and *G/R*-values at different oscillation cycles are presented in Figure 11. Closer to the tail of the molten pool, *G/R* tends to decrease, and *G×R* tends to increase. Depending on the pool shape, the calculated *G/R* in P_1_ to P_3_ decreased sharply compared to P_4_. Additionally, P_1_ and P_4_ maintained high *G×R*-values where the *y*-axis was more than 0.6 mm away from the center. Since *G×R* determines the grain size, it can be inferred that the grains formed at the tail in P_1_ and P_4_ will be coarser than those in P_2_ and P_3_. The *G* and *R* structure map is presented in Figure 12, highlighting the CET boundary (*G/R* = 3 K/mm). As the molten pool stretched (at P_1_ to P_3_), an equiaxed structure transition was observed at high R. In the P_3_ to P_1_ period, as the molten pool contracted, the equiaxed structure transition occurred at a relatively low *R*.

**Figure 13:**
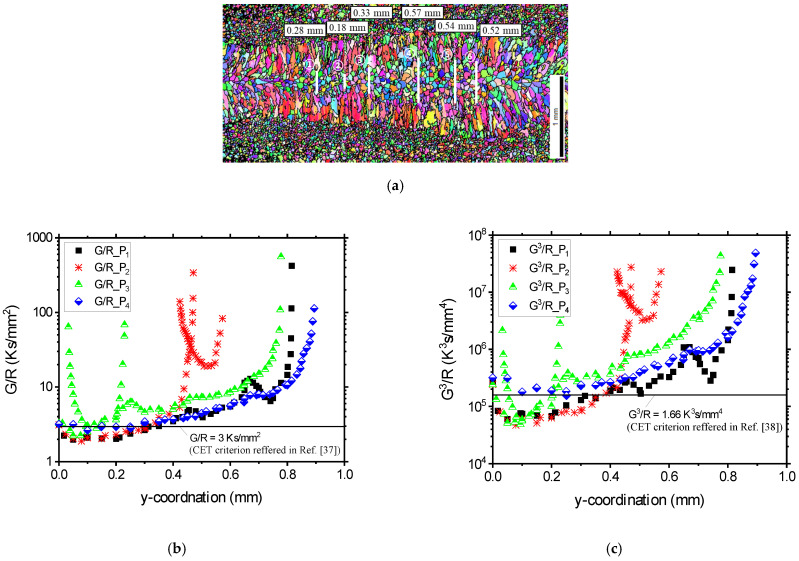
Comparison of actual and calculation results: (**a**) EBSD images in view of normal direction, calculated columnar to equiaxed structure transition threshold based on (**b**) *G/R* [37], and (**c**) *G*^3^/*R* [38].

The *G/R* and *G*^3^/*R* thresholds were used to predict the microstructural transition and were then compared with EBSD results (Figure 13). The CET boundary predicted using the *G/R* threshold was positioned 0.3 mm away from the center, while the *G*^3^/*R* threshold indicated it at 0.4 mm from the weld center. In comparison with the EBSD results, the *G/R* threshold showed greater consistency with the actual outcome. Therefore, it is expected that the capability to predict CET based on *G* and *R*, calculated through simulations under complex welding conditions such as oscillation welding, will aid in designing a process to create a fine equiaxed structure, thereby preventing hot cracks.

## 5. Conclusions

In this study, laser beam oscillation welding, characterized by a 25 Hz frequency and 1 mm width, was conducted and investigated through FEM-based CHT analysis. This approach effectively calculated temperature gradients and solidification rates, enhancing the understanding of molten pool dynamics and their influence on microstructural evolution during welding. Key findings are summarized below.

1. Observations from high-speed camera images indicated that the changing dimensions of the molten pool, driven by keyhole movement, critically affected the solidification rate and temperature gradient.
2. Although the simulated temperature gradient and solidification rate values were higher than those reported in the literature, the trends in solidification indices such as *G/R*, *G×R*, and *G*^3^/*R* were consistent with previously published findings.
3. The application of *G/R* and *G*^3^/*R* thresholds enabled accurate predictions of transitions from columnar to equiaxed structures. The *G/R* threshold, in particular, aligned more closely with EBSD observations compared to *G*^3^/*R*.

Consequently, this study offers significant insights into the control of temperature gradients and solidification rates in laser beam oscillation welding, pointing towards methodologies for inducing refined equiaxed structures and mitigating hot cracking through calculated *G* and *R*-values.

## Figures and Tables

**Table 1:** Selected laser welding parameters.

Parameter	Value
Laser power (kW)	3
Travel speed (m/min)	6
Focal position (mm)	0
Beam irradiating angle (°)	90
Beam pattern (-)	Lateral
Oscillation width (mm)	1
Oscillation frequency (Hz)	25
Shielding gas (-)	No

**Table 2:** Applied mechanical and thermal properties of Al 6061.

Properties	Unit	Value	Ref.
Density	kg/m^3^	2700	[31]
Latent heat	J/kgK	395,000	[32]
Thermal conductivity (S)	J/mK	*	[32]
Thermal conductivity (L)	J/mK	*	[32]
Specific heat (S)	J/kgK	*	[32]
Specific heat (L)	J/kgK	*	[32]
Liquidus temperature	K	927	[31]
Solidus temperature	K	889	[31]

Note: * indicates temperature-dependent data.

## Data Availability

Data are contained within the article.
